# Tectonic lamellar keratoplasty with acellular corneal stroma in high-risk corneal transplantation

**Published:** 2011-07-15

**Authors:** Naiyang Li, Xiaoran Wang, Pengxia Wan, Minghai Huang, Zheng Wu, Xuanwei Liang, Ying Liu, Jian Ge, Junqi Huang, Zhichong Wang

**Affiliations:** 1 State Key Laboratory of Ophthalmology, Sun Yat-sen University, Guangzhou, P.R. China; 2 Zhongshan Ophthalmic Center, Sun Yat-sen University, Guangzhou, P.R. China; 3 Department of Immunology, Institute of Immunology, Zhongshan School of Medicine, Sun Yat-sen University, Guangzhou, P.R. China

## Abstract

**Purpose:**

Tectonic lamellar keratoplasty (TLKP) is a primary surgical procedure to improve the condition of the recipient bed in high-risk corneal transplantation. It is usually performed for a secondary optical penetrating keratoplasty (PKP). The present study was undertaken to explore a new strategy for TLKP using acellular corneal stroma (ACS) prepared by decellularization.

**Methods:**

ACS for TLKP was prepared from cat cornea by decellularization. The efficiency of the decellularization was examined by hematoxylin and eosin (H&E) staining and through DNA content analysis. Twenty-eight New Zealand white rabbits, as recipients, were assigned to one of two groups that had different material for their TLKP. The TLKP was combined with a central optical PKP as a single-stage procedure. Either ACS or fresh cat corneal lamella, 11.25 mm in diameter, was used for the TLKP in these two groups. After TLKP, a 6.5-mm full-thickness cat cornea was placed in the central cornea of each recipient rabbit for PKP. Clinical outcomes and the histology of the transplants were compared post-operatively.

**Results:**

ACS for TLKP prolonged the survival of the transplants. The mean survival time of the transplants in the ACS group (36.4±4.3 days) was longer than for those in the control group (14.0±2.2 days, p<0.05). The ACS group showed a significantly smaller neovascularization area compared to the control group. The areas of corneal neovascularization were 5.3±1.1 mm^2^ and 45.2±4.9 mm^2^ (p<0.05), respectively, after two weeks, and 25.1±4.7 mm^2^ and 105.3±12.4 mm^2^ (p<0.05), respectively, after four weeks. Histology revealed that fewer inflammatory cells were infiltrating the transplants in the ACS group than those in the control group.

**Conclusions:**

The use of ACS for TLKP prolonged the survival of corneal transplants, reduced corneal neovascularization, and prevented from infiltration of inflammatory cells. It is a feasible and effective strategy to prolong the survival of transplants in high-risk corneal transplantation.

## Introduction

Corneal transplantation, including lamellar keratoplasty (LKP) and penetrating keratoplasty (PKP), is the only definitive treatment for corneal blindness [1-3]. It has a survival rate of 90% when performed in low-risk recipients [4]. Its high success rate can be attributed to the immune privilege of the cornea, including the absence of blood and lymphatic vessels in the cornea, the low expression of major histocompatibility (MHC) class I and II antigens on corneal cells, and anterior chamber-associated immune deviation [5]. However, the survival of transplants depends upon the condition of the recipient corneal bed [6]. In high-risk corneal recipients, such as those with inflamed or vascularized recipient beds and large-diameter or eccentric transplants, the immune privileges of the corneas are broken. In these cases, the survival rates of transplants fall to below 50%, even with immune-suppression therapy [7-11].

Tectonic lamellar keratoplasty (TLKP) is a useful strategy for improving the condition of the recipient corneal beds, to prolong the survival of transplants in the high-risk recipients [12,13]. It achieves this by removing inflamed or vascularized corneal recipient beds and replacing them with a large-diameter LKP. This procedure is usually used as a primary surgical procedure for a secondary optical PKP in patients with extensive peripheral corneal thinning, scarring, or neovascularization because of chemical or thermal burns. TLKP can partially restore the immune privilege of cornea and prolong the survival of secondary optical PKP in high-risk recipients [14].

However, there is a worldwide shortage of donor corneas [3,15]. To address this shortage, xenogeneic corneal extracellular matrix (ECM), or acellular corneal stroma (ACS), is developed for corneal tissue engineering through decellularization. The decellularization process could remove the cell components of the xenogeneic cornea while retaining its natural ECM. It could function as a constructive scaffold in mammals, without inciting a destructive inflammatory reaction, because the components of the ECM are highly conserved across species. Furthermore, this ECM could promote cell growth and be remodeled easily in vivo [3,15-20]. In our previous study, we found that ACS was a highly suitable material for LKP, due to its low immunogenicity, high transparency and favorable biocompatibility [21,22]. These findings prompted us to explore the potential further application of ACS. Since ACS was a suitable material for LKP, we hypothesized that it could be used in TLKP for low immunogenicity. The purpose of this study was to explore a new strategy for TLKP using ACS in a cat-rabbit model. The feasibility and effectiveness of this strategy are discussed.

## Methods

### Animals

The Institutional Animal Care and Use Committee of the Zhongshan Ophthalmic Center, Sun Yat-sen University, Guangzhou, P.R. China, approved this study. All experimental procedures were conducted in accordance with the Association for Research in Vision and Ophthalmology’s resolution on the use of animals in research. Twenty-eight New Zealand white rabbits (either gender, aged 10 weeks, and weighing 2–3 kg) were used as recipients. Twenty-one adult domestic cats (either gender, aged 4 years, and 3–5 kg) were used as donors for orthotopic corneal transplantation. The animals were housed under a 12-h:12-h light-dark cycle with standard food and water adlibitum.

### Preparation of ACS

Whole eyes (n=38) from 19 cats were obtained within 1–3 h after they were sacrificed. The 300-μm-thick anterior lamellae were prepared from cat corneas using an 11.25-mm-diameter trephine (Kai Industries Co., Seki City, Japan) and a crescent knife (Alcon, Fort Worth, TX). The lamellae (n=28) underwent a decellularization process as described in our previous report [21]. Briefly, a bicarbonate-mixed salt solution consisting of 105 mM NaCl, 5.5 mM KCl, 1.8 mM CaCl_2_, 0.8 mM MgCl_2_, 5 mM glucose, and 35 mM NaHCO_3_ (pH 8.3, 322 mOsmol/kg) was prepared. The lamellae were then immersed in the bicarbonate-mixed salt solution, which contained phospholipase A_2_ (200 U/ml) and 0.5% (w/v) sodium deoxycholate (all from Sigma-Aldrich, St. Louis, MO) for 6 h at 37 °C and subsequently immersed in bicarbonate-mixed salt solution containing phospholipase A_2_ (200 U/ml) for 2 h at 37 °C. All steps were conducted with continuous shaking in a thermostat-controlled water bath. Four prepared ACSs were randomly picked and embedded in paraffin for hematoxylin and eosin (H&E) staining to examine the decellularization efficiency under a light microscope (Carl Zeiss, Oberkochen, Germany). 11.25-mm-diameter central corneal discs of naive cat corneas and ACSs (n=10) were dried to constant weight in a cabinet drier at 37 °C for 72 h, and weighed. The samples were digested with 125 mg/ml papain solution (Sigma-Aldrich) at 60 °C for 24 h. The DNA content of the sample was determined by measuring the fluorescence (358/485 nm) of the aliquots of the digested mixtures with Hoechst 33258 (Invitrogen, Carlsbad, CA). The other ACSs were dried in a cabinet drier at 37 °C for 72 h. Then they were sealed in a plastic envelope, sterilized using γ-irradiation (25 kGy).

### Surgical procedures

**Figure 1:**
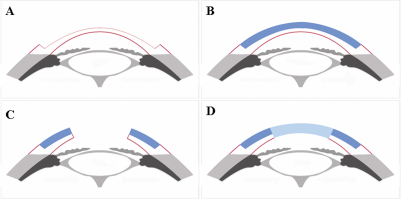
Schematic diagram of corneal transplantation procedure. **A**: The anterior lamella was dissected from the rabbit cornea. **B**: The prepared fresh cat corneal lamella/ACS were sewn into place for TLKP. **C**: The graft bed for PKP was prepared in the central cornea. **D**: A full-thickness donor cat cornea was placed in the recipient bed.

Twenty-eight rabbits were randomly assigned to one of two groups. ACS was used for TLKP in the ACS group and fresh cat corneal lamella was used for the control group. TLKP was combined with a central PKP as a single-stage procedure. The central PKP used full-thickness cat cornea. Orthotopic corneal transplantation was performed following the procedure described previously [23]. General anesthesia was through intramuscular injection of ketamine (25 mg/kg) and chlorpromazine (25 mg/kg). In the ACS group, the ACSs from sterile plastic envelopes were soaked in phosphate buffer saline for 10 min to recover tenacity (n=14). In the control group, the 300-μm–thick fresh cat corneal anterior lamellae were prepared from the cat corneas using an 11.25-mm–diameter trephine and a crescent knife (n=14). A 200-μm–thick and 11.0-mm–diameter anterior lamella was dissected from the rabbit cornea to generate the recipient bed (Figure 1A). The prepared ACSs/fresh cat corneal anterior lamellae were then sewn into the recipient bed for TLKP using 16 interrupted 10–0 nylon sutures (Figure 1B). After TLKP, the transplant bed for PKP was prepared in the central cornea of the rabbit using a 6.25-mm trephine (Figure 1C). A 6.5-mm transplant from the donor cat was placed in the recipient bed and secured with six-to-10 interrupted 10–0 nylon sutures (Figure 1D). The sutures passed through the posterior lamella of the recipient rabbit’s cornea. The two transplants for PKP and TLKP in the control group were from eyes of the same donor cat. Heparin (1,000 U/ml) was applied topically to prevent the formation of aqueous clots in the anterior chamber during the surgery. All rabbits received antibiotic eye drops four times daily after surgery for two weeks. When the suture was loosened after surgery, it was removed under topical anesthesia.

### Assessment of transplant survival

After surgery, corneal transplants in the central cornea were evaluated using a slit lamp every other day for two weeks and twice a week after that. A standard scoring system, using a 0-to-5 scale for corneal opacity, was adopted: 0=clear, 1=minimal superficial opacity, 2=mild stromal opacity with good visualization of the pupil margin and iris structures, 3=moderate stromal opacity with poor visualization of the iris structures, 4=intense stromal opacity with the anterior chamber visible, and 5=maximal corneal opacity with total obscuration of the anterior chamber. Transplants were considered rejected if a score of three or more was observed for two consecutive days, with no clearance [1]. Rabbits in both groups were followed for six weeks.

### Corneal neovascularization

Measurements of neovascularization were made with a slit lamp by a single observer blind to the group assignments. Neovascularization was quantified by calculating the wedge-shaped area of vessel growth, using the formula: A=C/12×3.1416[r^2^×(r-l)^2^], where A is the area, C is time (in hours), r is the radius of the cornea, and l is the radius from the center to the border of vessel growth [24].

### Histology and immunohistochemistry

At two and four weeks after transplantation, two rabbits from each group were sacrificed by air embolism. Their corneas were divided into two. One half was stained with H&E for histological examination. The other was embedded with an optimal cutting temperature compound (Sakura Finetechnical Co., Tokyo, Japan) and cut into 5-μm-thick slices. Monoclonal mouse anti-rabbit CD4 and CD8 were used as the primary antibodies (1:100; Invitrogen). The secondary antibody was Alexa Fluor 594 goat anti-mouse IgG (1:100; Invitrogen). Hoechst 33258 (1:2,000; Invitrogen) was used to detect nuclei, and the sections were viewed using a laser-scanning confocal microscope (Carl Zeiss, Oberkochen, Germany) [25].

### Statistical analysis

Data were expressed as mean±standard deviation and compared by Student’s *t*-test. Corneal transplant survival data were plotted with a Kaplan–Meier curve. A p value of <0.05 was considered statistically significant (SPSS 12.0; SPSS, Chicago, IL).

## Results

### Decellularization efficiency

**Figure 2:**
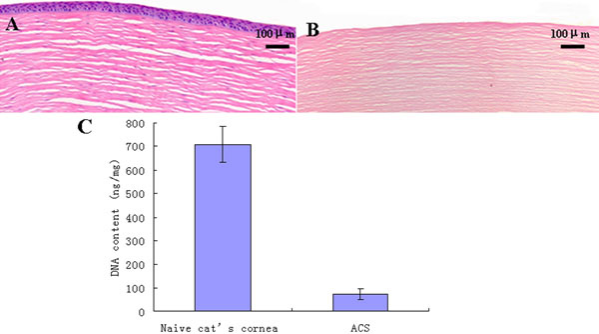
Efficiency of decellularization. **A**: A naive cat cornea. **B**: No visible corneal cells were observed in ACS by H&E staining. **C**: More than 90% DNA content of a naive cat cornea was removed by decellularization process (from 710.3±75.3 ng/mg to 70.6±22.5 ng/mg, n=10, p<0.05).

Corneal cells in each layer were removed by decellularization process. No visible cells were observed in the ACS by H&E staining. The stromal layers were arranged tightly and fibrils appeared orderly. The amount of DNA of the naive cat cornea and the ACS were 710.3±75.3 ng/mg and 70.6±22.5 ng/mg dry weight, respectively (n=10, p<0.05; Figure 2).

### Clinical course and survival of corneal transplants

**Figure 3:**
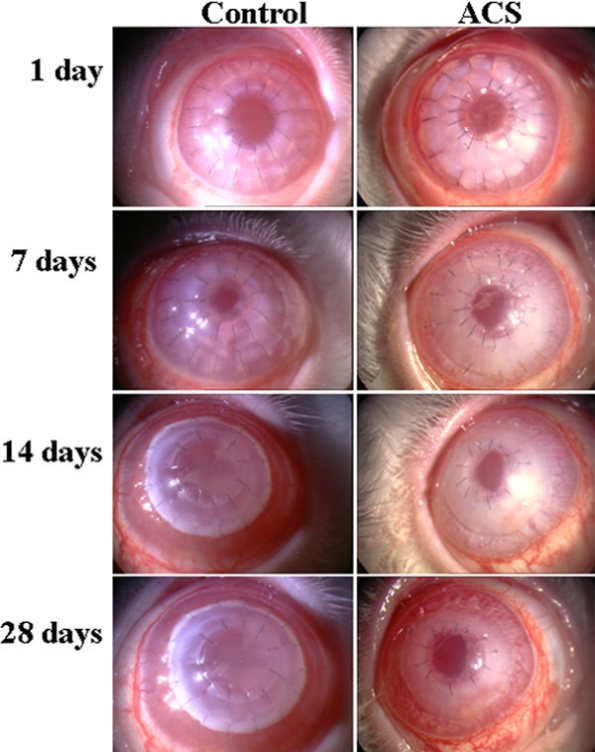
Clinical course of transplants in the cat-rabbit model. Immune rejection occurred earlier and more severely in the control group than in the ACS group.

**Figure 4:**
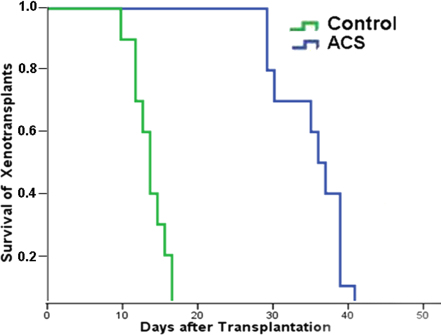
Kaplan–Meier survival plot of transplants. ACS for TLKP significantly prolonged the central transplant survival (n=10, p<0.05).

Slight corneal edema was seen in all rabbits after surgery, but disappeared within two days in both groups. All transplants in the center remained transparent during the first post-operative week. In the control group, corneal edema started to develop eight-to-10 days after surgery, and the transplants subsequently showed signs of immune rejection, with loss of transplant clarity, increased edema, and progressive neovascularization. All transplants were completely rejected in the control group by 17 days after surgery. In the ACS group, the re-epithelialization time of ACS in the peripheral cornea was 3±0.6 days (n=14). After re-epithelialization, the ACS turned transparent. The central transplants remained transparent for 29-to-32 days. The transparency of the central transplants decreased thereafter, and corneal edema occurred (Figure 3). The mean survival time of the central transplants in the ACS group (36.4±4.3 days) was longer than in the control group (14.0±2.2 days; n=10, p<0.05; Figure 4).

### Corneal neovascularization

**Figure 5:**
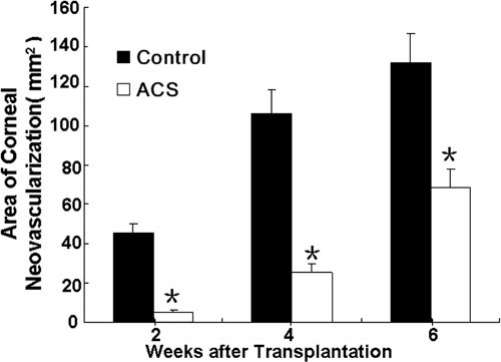
Area of corneal neovascularization. The mean area in the ACS group was fewer than control group 2 and 4 weeks post-operatively (n=10, p<0.05). The asterisk indicates a p<0.05.

Corneal neovascularization in the ACS group occurred later than in the control group. In the latter group, new vessels started to grow toward the central transplants from the limbus six days after the surgery. The amount of neovascularization then increased and remained at a high level. In the ACS group, new vessel development occurred 21-to-28 days after transplantation, and this group showed a significantly smaller neovascularization area compared to the control group. The areas of corneal neovascularization in the ACS and control groups were, respectively, 5.3±1.1 mm^2^ and 45.2±4.9 mm^2^ (n=10, p<0.05) two weeks after surgery and 25.1±4.7 mm^2^ and 105.3±12.4 mm^2^ (n=10, p<0.05) four weeks after surgery (Figure 5).

### Histology and immunohistochemistry of corneal transplants

**Figure 6:**
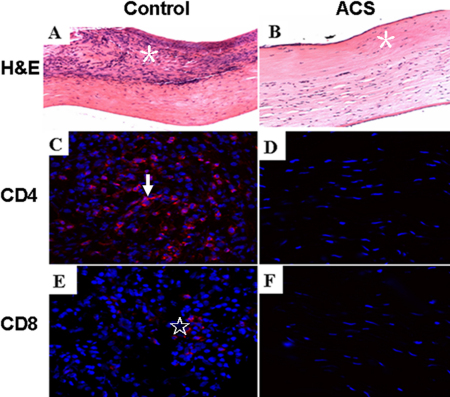
H&E and immunofluorescence staining of transplants 2 weeks after surgery. In the control group, transplants were infiltrated with a large number of inflammatory cells (**A**, asterisk), CD4+ (**C**, arrow) and CD8+ T cells (**E**, star); fewer inflammatory cells (**B**), CD4+ (**D**) and CD8+ T cells (**F**) were observed in the ACS group. (**A**, **B**; 200×, **C**-**F**; 400×).

In the control group, histological examination revealed that the transplants were infiltrated with a large number of inflammatory cells and newly formed blood vessels entered the transplants two and four weeks after transplantation. The inflammatory cells were mainly concentrated in the anterior lamellae, with fewer observed in the posterior lamellae (Figure 6A). In the ACS group, fewer inflammatory cells and new vessels were observed in the transplants two and four weeks after surgery (Figure 6B).

Immunohistochemical staining showed abundant CD4+ and CD8+ T cells infiltrating the transplants in the control group, but not as many CD4+ and CD8+ T cells were observed in the ACS group (Figure 6C-F).

## Discussion

Corneal transplant rejection involves donor antigens from the transplant being processed by antigen-presenting cells (APCs) and presented to the transplant host naive T cells. Reactive cells and molecules then move to the transplant and attack it. The survival of transplants depends on the condition of the recipient corneal bed. For high-risk recipient corneal beds, the transplant is close to blood or lymphatic vessels, which helps donor antigens recognize, present and transport reactive cells or molecules, and to attack the transplant. This situation breaks corneal immune privilege and accelerates the immune rejection [6,26-28]. To improve the recipient bed conditions and restore immune privilege, TLKP is a useful strategy as a primary surgical procedure because it can remove corneal scarring and new vessels. A secondary optical PKP to obtain visual rehabilitation can be performed after TLKP.

In this study we explored a new strategy for TLKP, using ACS, in a cat-rabbit model. TLKP was combined with a central optical PKP as a single-stage procedure. First, a large-diameter TLKP using ACS was performed to improve the recipient bed for PKP; then, a central optical PKP was performed to restore corneal transparency on the improved bed. We found that, in this high-risk corneal transplantation model, the transplants in the ACS group survived longer (36.4±4.3 days) than those in the control group (14.0±2.2 days). ACS for TLKP prolonged corneal transplant survival by reducing neovascularization and the infiltration of inflammatory cells.

There are two main reasons for the prolonged the survival of transplants using ACS for TLKP. First, ACS prepared by decellularization has lower immunogenicity and less ability to induce immune rejection. The decellularization removes the cell components, while retaining its ECM. The amino acid sequence and quaternary structure of ECM are highly conserved across the mammal species. Because of this relationship, xenogeneic ECM can function as a constructive scaffold without inciting a destructive inflammatory reaction [3,16]. Moreover, native ECM has innate biologic cues, which help to direct and organize cell function, including cell differentiation, proliferation, and migration [29]. Native ECM has been developed as a biologic scaffold for tissue engineering applications in the vessel, heart valve, dermis, ligament, etc [3,15,30]. In our study, ACS derived from xenogeneic corneas had lower immunogenicity and less ability to induce neovascularization and inflammatory cell infiltration than did fresh cat corneal lamellae. Thus, there were fewer corneal neovascularization and CD4+ and CD8+ T cells in the ACS group than in the control group. Another reason why TLKP using ACS prolonged the survival of transplants is that ACS partially blocks both afferent and efferent corneal immune rejection. Blood vessels and lymphatic vessels in the limbus assist donor antigens in recognizing, presenting and transporting reactive cells or molecules, and attacking the transplant. In this study, ACS for TLKP isolated the central transplant from the limbus and increased the distance between it and the central transplant. Moreover, ACS had no blood vessels, no lymphatic vessels, no APC, and even no nerve fibers in a short time [21,31]. Yamagami and Dana [32] found that surgical severing of the lymphatic pathway led to universal corneal transplant survival without any form of pharmaceutical immune modulation. Therefore, ACS for TLKP slowed the immune response process and prolonged the survival of corneal transplants.

In our study, corneal neovascularization had a close relationship with immune rejection. Corneal neovascularization occurred later and less frequently in the ACS group than in the control group. Neovascularization of the corneal transplant may be the result of a poorly prepared recipient eye and a sign of immune reaction [33]; the new vessels broke immune privilege and brought more immune molecules, which accelerated the immune response process [11,34].

In clinical practice, ACS for TLKP has two key advantages. It can avoid both repeated surgical procedures and large-size PKP. First, repeated surgical procedures can result in iatrogenic injury and increase the risk of immunologic rejection due to the recipient being pre-sensitized [35]. In our study, because ACS for TLKP was combined with a central optical PKP as a single-stage procedure, iatrogenic injury could be avoided and the incidence of rejection associated with repeated surgical procedures reduced. Second, although large-diameter PKP can successfully recover corneal thickness and remove full-thickness corneal opacity after injuries from chemical or thermal burns, both of which are common indications for corneal transplantation in China [13], it has a high incidence of rejection. Moreover, many postoperative complications may occur through the collapse of the trabecular meshwork and peripheral anterior synechiae [9]. A large-diameter TLKP using ACS can recover the thickness of the cornea and provide an improved bed for PKP, for a central optical PKP to be performed to restore corneal transparency. As such, the strategy can avoid large-sized PKP.

The corneal xenotransplation model contains a high-risk for immune rejection. In this cat-rabbit model, we observed typical rejection reactions characterized by neovascularization and opacification of the transplants. However, unlike the neovascularization model induced by silk sutures or alkali burn before transplantation, this xenotransplantion model saves time and avoids tedious procedures.

### 

#### Conclusions

In conclusion, we explored a new strategy for TLKP using ACS. TLKP was combined with a central optical PKP as a single-stage procedure in a cat-rabbit model. It prolonged the survival of corneal transplants through less vessel growth and inflammatory cell infiltration. Therefore ACS for TLKP is both a feasible and an effective strategy to prolong the survival of transplants in high-risk corneal transplantation.
